# Prevalence and risk factors of *Toxoplasma gondii* infection among women with miscarriage and their aborted fetuses in the northwest of Iran

**DOI:** 10.1371/journal.pone.0283493

**Published:** 2023-10-26

**Authors:** Shiva Zeinali, Shahram Khademvatan, Rasool Jafari, Shabnam Vazifekhah, Elham Yousefi, Tahereh Behroozi-Lak

**Affiliations:** 1 Department of Medical Parasitology and Mycology& Cellular and Molecular Research Center, Cellular and Molecular Medicine Research Institute, Urmia University of Medical Sciences, Urmia, Iran; 2 Maternal and Childhood Obesity Research Center, Urmia University of Medical Sciences, Urmia, Iran; 3 Reproductive Health Research Center, Department of Infertility, Urmia University of Medical Sciences, Urmia, Iran; Mashhad University of Medical Sciences, ISLAMIC REPUBLIC OF IRAN

## Abstract

Toxoplasmosis is a worldwide disease of various animals and human and results from infection with the *Toxoplasma gondii* parasite. Abortion and congenital defects are important consequences of the *T*. *gondii* infection. The aim of this study was to determine the *Toxoplasma*-induced abortions among women with miscarriage and the presence of *T*. *gondii* in their aborted fetuses in Urmia, the northwest of Iran. This cross-sectional study was conducted with 215 women with abortion and their aborted fetuses, from 2020 to 2021. Seroprevalence of anti-*Toxoplasma* IgG and IgM were determined using the sera of the aforesaid women. Nested PCR was carried out using *RE-529* gene sequences, and sequencing was performed using the *T*. *gondii GRA6* gene on the remnant of pregnancy after abortion. The tissue positive samples were then subjected to another PCR on *GRA6* gene and sequenced for genotyping. Among 215 serum samples of women with abortion, 70 (32.6%) were positive for anti-*Toxoplasma* IgG, and three (1.4%) were positive for IgM. The *RE-529* sequence of *T*. *gondii* was positive in three (1.4%) of the aborted fetuses. The analysis of *GRA6* gene indicated that all three positive samples carried a *GRA6* allele (*GRA6I*) of *T*. *gondii* type I genotype. Our findings suggest that *T*. *gondii* is one of the causative agents of spontaneous abortion in West Azerbijan Province, the northwest of Iran.

## 1. Introduction

Miscarriage or spontaneous abortion is one of the most frequent concerns of pregnant women around the world [1]. An early miscarriage happens during the first 12 weeks of gestation, and a late miscarriage occurs between 12 and 24 weeks [2]. About 10–30% of all miscarriages have an infectious etiology; 5% of early abortions and 66% of late abortions are related to *Toxoplasma gondii* infections [3]. However, the low rates of diagnostic testing for infections in pregnancy may lead to the underestimation of abortion incidence. The rate of pregnancy loss depends on gestational age at infection and pathogen type [4].

Toxoplasmosis is a parasitic infection induced by protozoan *T*. *gondii* and has various clinical manifestations [5]. The parasite is broadly distributed worldwide and can infect a broad range of warm-blooded organisms, including humans, pets, and livestock [1, 6]. In the general population, *T*. *gondii* infection can remain asymptomatic and cause lymphadenopathy and flu-like symptoms, but in immunecompromised patients, it can be fatal [1]. In pregnant women, the parasite may cause infection in the fetus. When a pregnant woman ingests *Toxoplasma* oocysts or tissue cysts for the first time during pregnancy, tachyzoites spread throughout the body via the blood [7]. In this stage, known as acute toxoplasmosis, the parasite can cross the placenta and infect the fetus. The risk of transmission after primary infection varies from 25% (in the first trimester) to 65% (in the last trimester) [8]. Thus, the risk of fetal infection in the first 13 weeks of pregnancy is about 15%, which increases rapidly after the mentioned time [9]. In the fetus and infant, congenital toxoplasmosis (CT) can give rise to various complications such as abortion, stillbirth, and live birth of a child with the classic symptoms of toxoplasmosis viz hydrocephaly, microcephaly, cerebral calcifications, and retinochoroiditis [10]. The global prevalence rate of CT is 190,100 cases annually with an approximate incidence rate of 1.5 cases per 1,000 live birth [11]. In Iran, the frequency of toxoplasmosis in pregnant women is estimated as 41% [12]. The early diagnosis of the onset of the infection and the timely diagnosis of acute toxoplasmosis during pregnancy is crucial for the protection of mother and fetus, as well as the assessment of vertical transmission risk of the infection [13]. Therefore, specific treatment of mothers can reduce the risk of fetal infection up to 50% and prevent related severe complications [14].

Serological tests are common diagnostic methods for CT. However, these techniques sometimes fail to detect particular anti*-T*. *gondii* antibodies during the early phase of the infection. Molecular diagnostic methods have been shown to be more sensitive and specific than serological tests for diagnosing CT [15]. Genetic analysis of *T*. *gondii* suggests the existence of different variants, being highly clonal and exhibiting a low genetic diversity. These variants include strains type I, II, and III, as well as Africa I [5, 16, 17]. There is also a great variety of *T*. *gondii* genotypes with different pathogenicity in CT cases. In addition to parasite burden and gestation period, *T*. *gondii* genotype seems to be involved in the clinical consequences of CT [18]. In this study, we aimed to determine the prevalence of toxoplasmosis in women with miscarriage and the presence of *T*. *gondii* in their aborted fetuses using serological and molecular approaches in Urmia, the northwest of Iran, from 2020–2021.

## 2. Materials and methods

### 2.1. Ethical statement

This study was approved by the Ethics Committee of Urmia University of Medical Sciences, Urmia, Iran (Ethical code: IR.UMSU.REC.1399.078). All the patients gave their signed written informed consents before the initiation of the study.

### 2.2. Sample collection

**Fig 1:**
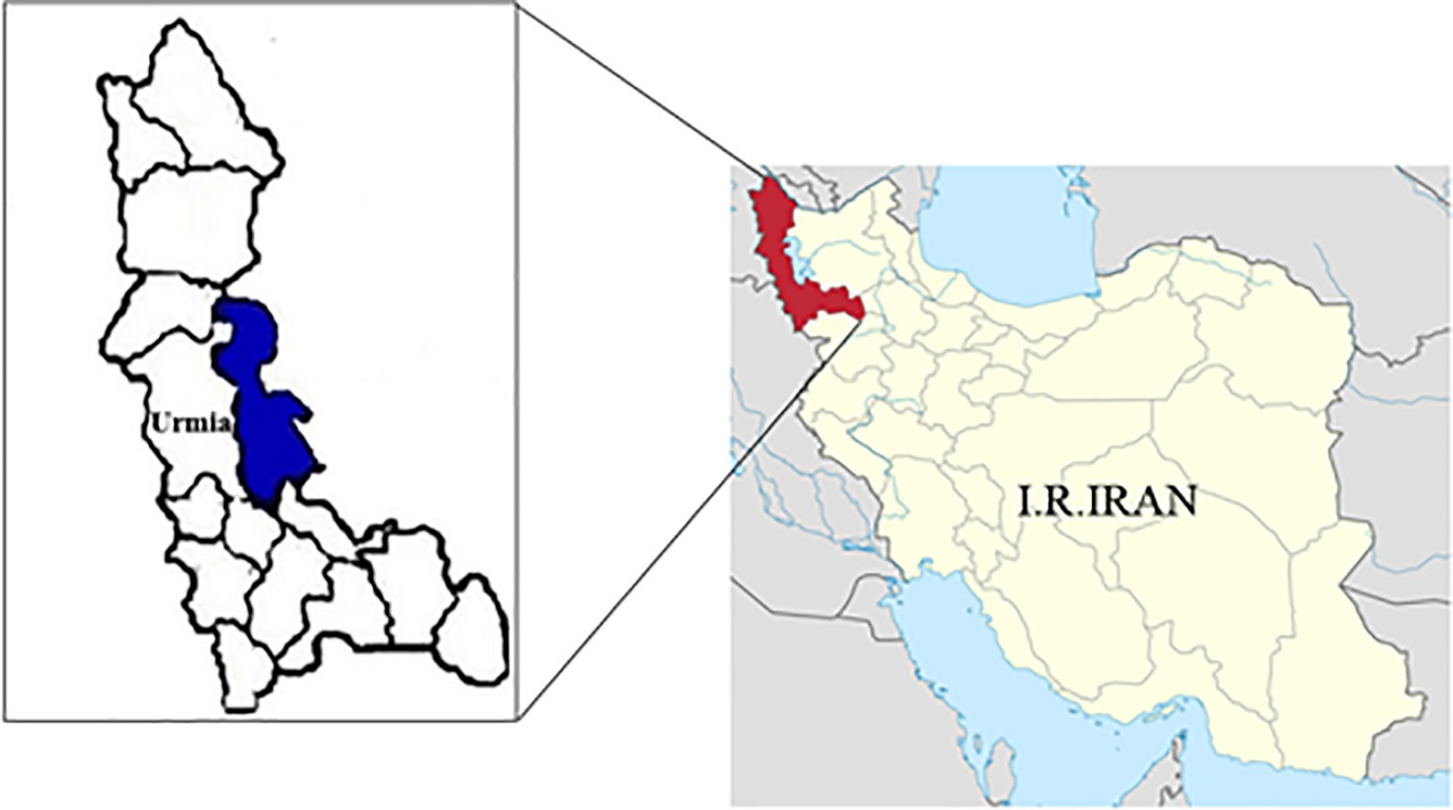
The study area on the map of Iran. The West Azerbaijan Province, Lake Urmia, and divisions of the province are depicted in red, blue, and white color, respectively.

The present cross-sectional study was carried out among female subjects (n = 215) who were referred to Motahari Hospital in Urmia, the northwest of Iran (Fig 1), from 2020 to 2021. Serum samples (n = 215) and aborted fetus samples (n = 215) were collected from all women studied. Demographic variables of the patients (women) with miscarriage are represented in Table 1 and include age, education, job, race, gestational age at abortion, history of previous abortion, place of residence, contact with cat and soil, consumption of undercooked meat and liver, and method of washing vegetables. The blood serum and abortion (pregnancy remnants) samples collected from all the patients were kept frozen at -80°C until serological tests and DNA extraction, respectively.

**Table 1:** Demographic variables associated with *T*. *gondii* seropositivity in pregnant women with spontaneous abortion (Pearson Chi-Square).

Variable	IgG	P	OR
	Positive n (%)		(Postive/Negative)
**Age group (year)**			
≤20	16 (61.5)	**0.006**	-
21–30	30 (26.5)		2.612
31–40	24 (32.0)		0.523
≥41	0		-
Total: 215	70 (32.6%)		
**Ethnicity**			
Azarbijani	34 (30.9)	0.571	
Kurdish	23 (31.5)		
Persian	13 (40.6)		
Total: 215	70 (32.6)		
**Job**			
Housewife	33 (28.7)	0.431	-
Worker	15 (36.6)		0.654
Employee	22 (37.4)		0.877
Total: 215	70 (32.6)		
**Education**			
Illiterate	7 (35.0)	0.604	
High school	21 (27.3)		
Diploma	17 (41.5)		
Associate degree	14 (30.4)		
Bachelor or higher	11 (35.5)		
Total: 215	70 (32.6)		
**Place of residence**			
Urban	38 (34.5)	0.312	0.681
Rural	32 (30.5)		-
Total: 215	70 (32.6)		
**Contact with cat**			
Indoor cats	5 (71.4)	0.001	-
Outdoor cats	56 (51.4)		0.037
No contact	9 (9.1)		0.233
Total: 215	70 (32.6)		
**Undercooked meat**			
Cooked	50 (25.9)	0.001	-
Uncooked	20 (90.9)		0.062
Total: 215	70 (32.6)		
**Undercooked liver**			
Cooked	44 (24.6)	0.001	-
Uncooked	26 (72.2)		0.203
Total: 215	70 (32.6%)		
**Contact with soil**			
Often	58 (52.3)	0.001	-
Occasionally	11 (12.6)		0.056
No contact	1 (5.9)		0.203
Total: 215	70 (32.6)		
**Washing vegetables method**			
Water	2 (66.7)	0.001	-
Salt	42 (55.3)		11.344
Disinfectants	26 (19.1)		3.603
Total: 215	70 (32.6)		

Odds ratio was estimated for positive IgG among different risk factors using binary logistic regression.

### 2.3. Serological analysis

Serum samples were tested for anti-*Toxoplasma* IgM and IgG using enzyme-linked immunosorbent assay (ELISA) kits according to the instructions provided by the manufacturer (Pishtaz Teb, Iran). The values higher than the index cut-off value of 1.1 (index) and less than 0.9 were considered positive and negative, respectively. Besides, the values between 0.9 and 1.1 were regarded to be equivocal and retested.

### 2.4. DNA extraction

Tissue DNA was extracted from pregnancy remnants using conventional phenol-chloroform extraction method based on the report from Sambrook et al. with modifications [19]. The procedure of the DNA extraction was briefly as follows: at first, the tissue was homogenized, and then 500 μl of lysis buffer (Tris-HCL, Nacl, SDS) and 20 μl of proteinase k were added to a tube containing the homogenized tissue. Subsequently, the suspensions were vortexed and incubated in a water bath at 56°C for 3 hours. Thereafter, a volume of phenol:chloroform:isoamyl alcohol mixture (25:24:1) was added to the lysate and centrifuged at 13,000 rpm for 10 minutes. The supernatants were then transferred to a new microtube, and an equal volume of chloroform was added to the microtubes and centrifuged again at the highest speed (13000 rpm). The latter process repeated for three times. The supernatants were then transferred to new microtubes, to which sodium acetate (10% of the volume) and cold isopropanol (an equal volume) were added. Microtubes were instantly kept at -21°C for 16 hours and centrifuged at 13,000 rpm for 30 minutes, and the supernatants were carefully discarded. The sediments (DNA) were washed several times with 70% ethanol (1 mL each time) for 6 minutes. After discarding the supernatants, microtubes were placed on a 50°C heater to dry out the excess liquid, then 50 μl of sterile nuclease-free water was added to each microtube, incubated for 3 minutes, and stored at -20°C until use.

### 2.5. Nested PCR

Nested PCR was employed to detect *T*. *gondii* DNA in the aborted fetuses’ tissues. The *RE-529* sequence of *T*. *gondii* was used for nested PCR using the external and internal primers. Outer primers (F: 5′-TGA CTC GGG CCC AGC TGC GT-3′ and R: 5′-CTC CTC CCT TCG TCC AAG CCTCC-3′) amplified a 420-bp region of *T*. *gondii*, and inner primers (F: 5′-AGG GAC AGA AGT CGA AGG GG-3′ and R: 5′-GCA GCC AAG CCG GAA ACA TC-3′) amplified a 164-bp region of round two [20]. The nested PCR reaction was performed on a final volume of 20 μL containing 10 μL of Master Mix (GeneDireX, Taiwan), 1 μL of each primer, and 6 μL of nuclease-free water. The reaction was carried out under the following conditions: 5 min hot start at 94°C, 35 cycles of denaturation at 94°C for 10 s, annealing at 55°C for 30 s, extension at 72°C for 30 s, and final extension at 72°C for 5 min. The round two PCR reaction was performed using the inner primers under the following conditions: 5 min hot start at 95°C, 35 cycles of denaturation at 95°C for 30 s, annealing at 55°C for 30 s, extension at 72°C for 30 s, and final extension at 72°C for 5 min. The PCR products were analyzed on 1.5% agarose gel electrophoresis [20]. The positive samples were subjected to another round of PCR on the 344-bp fragment of *T*. *gondii GRA6* gene in using the following pair of primers: forward: 5’-TTTCCGAGCAGGTGACCT-3’ and reverse: 5’- TCGCCGAAGAGTTGACATAG-3’ [21]. PCR carried out under the following conditions: 5 min hot start at 95°C, 35 cycles of denaturation at 95°C for 30 s, annealing at 60°C for 30 s, extension at 72°C for 35 s, and final extension at 72°C for 5 min. The PCR reaction (50 μL) consisted of 25 μL of Master Mix (GeneDireX), 1 μL of each primer, and 10 μL of nuclease-free water. The resultant amplicons were sequenced by the Sanger sequencing method and compared to the GenBank reference sequences using the BLAST tool (https://blast.ncbi.nlm.nih.gov/Blast.cgi). The obtained sequences were then calculated and an evolutionary tree was drawn by MEGA X [22] software by applying BioNJ and Neighbor-Join algorithms using the Tamura-Nei model [23].

### 2.6. Statistical analysis

All statistical analyses of the data were performed using SPSS (Ver.16) software (IBM, Armonk, New York, USA). The Chi-square test was employed to determine an association between seropositivity for *Toxoplasmosis* and qualitative variables. A *P* value less than 0.05 was considered as statistically significant.

## 3. Results

### 3.1. Characteristics of the study population

The age of women with spontaneous abortions ranged from 18 to 41 years old (mean: 28.15 ± 5.82). Abortion among the patients occurred in 55.3% in the first trimester and 44.7% in the second trimester of the prenatal period. Overall, 58.6% of the cases had no previous history of abortion.

### 3.2. Serological results

Anti-*Toxoplasma* antibodies were detected in sera of 73 (34%) out of 215 samples. Among these 73 samples, 70 (32.6%) were positive for anti-*Toxoplasma* IgG, while 3 (1.4%) were positive for anti-*Toxoplasma* IgM. Two (2.9%) cases were simultaneously seropositive for IgG and IgM. Of 70 IgG-positive cases, 30 (42.8%) had a history of previous abortion(s). There was a significant relationship between *T*. *gondii* IgG seropositivity and age (P = 0.006), consumption of undercooked meat and liver (P = 0.001), contact with cat and soil (P = 0.001), and the method of washing vegetables (P = 0.001). However, there was no significant correlation of anti-*T*. *gondii* IgG seropositivity with other studied variables. Because IgM seropositivity was observed in very few patients (n = 3), its relationship with risk factors was not discussed. Table 1 illustrates demographic variables related to IgG and IgM *T*. *gondii* seropositivity in pregnant women with spontaneous abortion.

Analysis of the data on the residential status of the patients demonstrated that the prevalence of IgG seropositivity was 30.5% (n = 32) and 34.5% (n = 38) in patients living in rural and urban areas (P = 0.312), while that of IgM seropositivity was 1.9% (n = 2) and 9% (n = 1) in these regions (P = 0.482), respectively. The prevalence of IgG seropositivity in different parts of the West Azerbaijan Province was 30.8% (n = 4) in the north, 33.0% (n = 62) in the center, and 28.6% (n = 4) in the south. However, the prevalence of IgM seropositivity was 1.6% (n = 3) in the center, and no positive case was detected in the north and south. The highest number of positive cases of IgG and IgM was reported as 33.0% and 1.6% in the center and 30.8% and 0 in the north of the aforementioned Province, respectively.

### 3.3. Molecular findings among aborted fetuses

**Fig 2:**
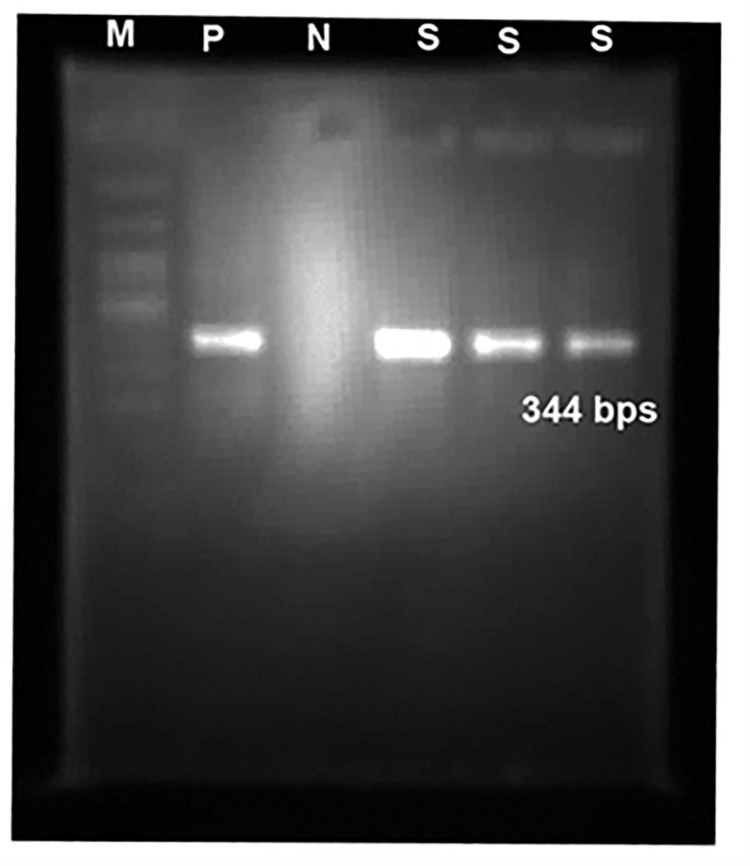
Agarose gel electrophoresis of *T*. *gondii GRA6* gene amplification. M, 100 bp molecular weight marker; P, positive control; N, negative control; lanes S, PCR product of 344-bp fragment of *T*. *gondii GRA6* gene.

All the samples were tested for *T*. *gondii* DNA using the nested PCR method. Among 215 samples, 3 (1.4%) were positive for *T*. *gondii* (Fig 2, and Table 2).

**Table 2:** ELISA and nested PCR results in samples.

Samples			Toxoplasma IgM		Total	Toxoplasma IgG		Total
			neg	pos		neg	pos	
PCR	Neg	Count %	212 100	0 100	212 98.6	144 99.3	68 97.1	212 98.6
							
	Pos	Count %	0 100	3 100	3 1.4	1 0.7	2 2.9	3 1.4
Total		Count %	212 100	3 100	215 100	145 100	70 100	215 100

Neg, negative; Pos, positive

### 3.4. *Toxoplasma* genotypes among aborted fetuses

**Fig 3:**
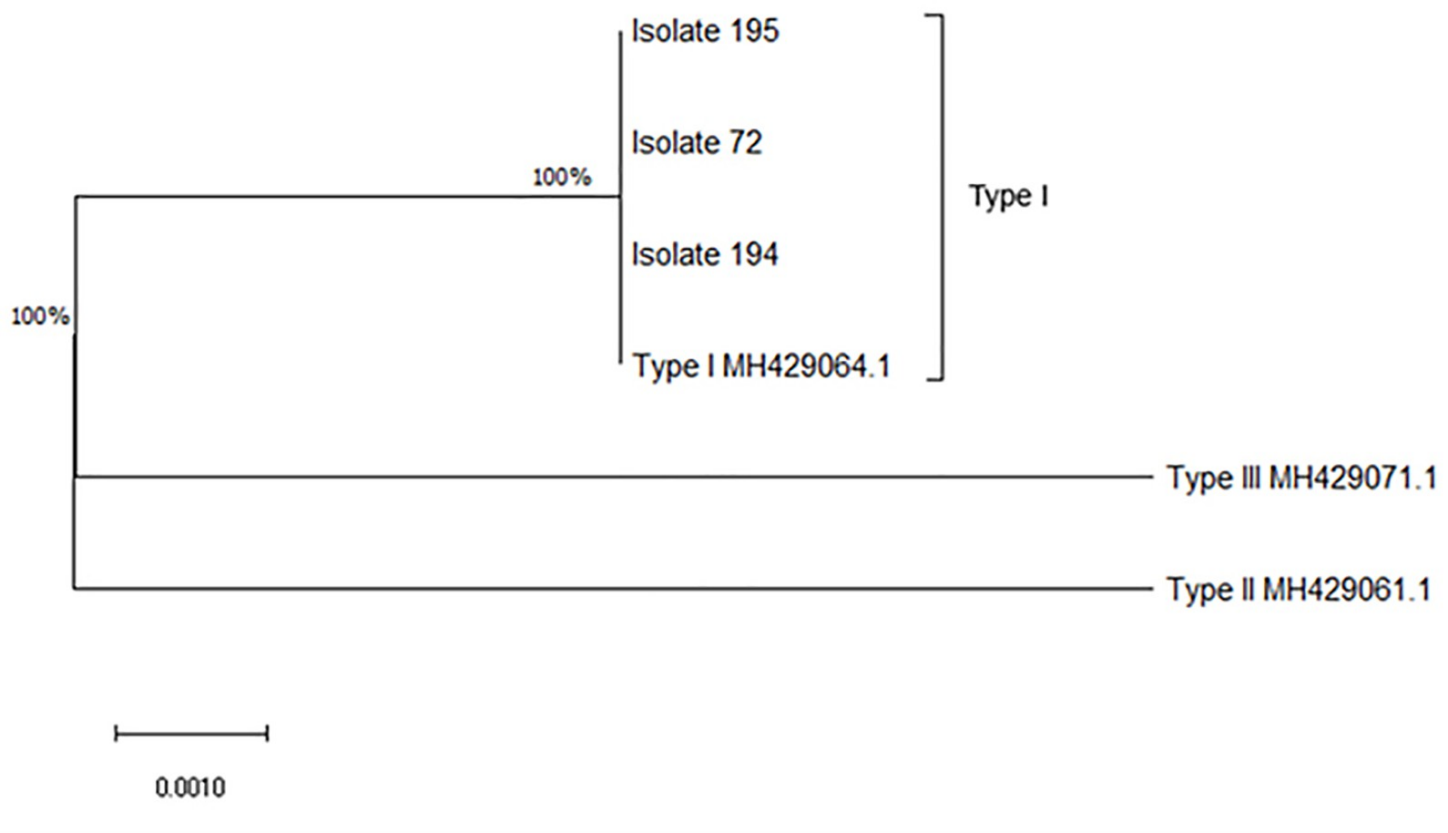
Evolutionary analysis of *T*. *gondii* isolates from aborted fetuses. Evolutionary analyses were conducted in MEGA X [22], and evolutionary history was inferred using the Maximum Likelihood method and Tamura-Nei model [23]. There was a total of 283 positions in the final dataset.

All three positive samples carried the *GRA6I (GRA6* allele of type I*)* with 100% homology to the accession number MH429064.1 [24]. The obtained sequences are available in supplementary file. Moreover, no polymorphism was identified in the studied sequences among the three *T*. *gondii* isolates, and all three positive PCR results were observed in the provincial center (Fig 3).

## 4. Discussion

The results of this study suggested that among 215 serum samples collected from women with abortion and their aborted fetuses, 70 (32.6%) were positive and 145 (67.4%) were negative for IgG, and only 3 (1.4%) samples were positive for IgM. In two investigations conducted in Iran on pregnant women, the seroprevalence of *T*. *gondii* IgG antibody was lower than our study, but that of IgM antibody was similar to ours in one of the studies and lower in another one [12, 25]. In some other surveys conducted in the country on women with spontaneous abortions, the seroprevalence of *T*. *gondii* IgG and IgM antibodies was reported to be 7% and 3% in Yasuj (in the southwest), 43% and 4% in Ardabil (in the northwest), 24.6% and 1% in Ahvaz (in the southwest), and 25.5% and 2.7% in Tehran, the capital of the country, respectively [8, 14, 26, 27]. However, our study showed higher seroprevalence of IgG and IgM antibodies than most of the above-mentioned research. In two studies conducted in Turkey and Iraq, the neighboring countries of Iran, the seroprevalence of IgG anti-*Toxoplasma* antibodies in women with spontaneous abortions was 30.6% and 6.6%, while that of IgM was 37.9% and 5.2%, respectively [28, 29]. In a global study, the seroprevalence of anti-*T*. *gondii* IgG and IgM antibodies in women with a history of abortion and with an abortion in the present pregnancy was reported as 43% and 3% and 33% and 1%, respectively [3]. In Iran, toxoplasmosis IgG and IgM antibodies in pregnant women was estimated to be 38% and 4%, respectively. In addition, the highest and the lowest seroprevalence of toxoplasmosis in five geographical zones of Iran was observed in the south (53%) and east (33%), respectively [12].

The prevalence of toxoplasmosis was significantly high in women of our study in terms of age and history of eating undercooked meat and liver, contact with cat and soil, and washing vegetables with water (P < 0.05; Table 1). IgG seropositivity was also significantly higher in patients who had a contact with soil than those who did not have (P < 0.001); As IgM seropositivity was observed in only very few patients (n = 3), its relationship with risk factors was not discussed. In supporting of our finding, studies conducted in Ahvaz and Yasuj, the southwest of Iran, found high seropositivity of IgG in women who had a contact with soil [14, 26]. A possible reason for the high seropositivity of IgG in the two aforesaid studies is that rural individuals have more contact with soil than urban people. In an earlier study conducted in Yasuj, half (50%) of the women with abortion had contact with cats, and only 8% had positive serology [26]. Also, in a study in Egypt, among 28 people who had contact with cats, 14 (50%) were seropositive, and 7 (25%) were positive in terms of real-time PCR [30]. In the study of Machumi et al. in Tanzania, a significant relationship was found between contact with cats and positive IgG serology [31].

In two surveys in West Azerbaijan Province, one in Urmia and one in Miandoab city, on pregnant women who kept cats at home or had a history of contact with cats, positive cases of IgG and IgM antibodies were identified, but the correlation was insignificant [12, 32]. In a further study conducted in Urmia city, the seroprevalence was not related to contact with cats [25]. In our study, 25.4% of patients who consumed undercooked, juicy, or raw meat was seropositive to IgG, and of these patients, 9.1% were seronegative. A potential reason for the high level of IgG seropositivity is the studied population may be the consumption of undercooked barbecue and liver. Moreover, 90.9% and 9.1% of women with abortion who consumed undercooked meat had IgM seropositivity and seronegativity, respectively (Table 1). These outcomes show a significant association between the consumption of undercooked, juicy, or raw meat and the positive cases of IgG, which supports the finding of Arefkhah et al. in the southwest of Iran [26]. There was also a significant correlation between the method of washing vegetables and both IgG and IgM positivity. In the study of Sharbatkhori in Gorgan city, the north of Iran, relationship between the method of washing vegetables and the infection rate of people who used these vegetables was nonsignificant [33]. Lack of hygiene and disinfectants for washing vegetables could be a cause of high IgG and IgM in that study.

Regarding risk factors of *T*. *gondii* infection, the risk of infection in people who did not have contact with cats was OR = 0.03, meaning that contact with cats outside the home 27 times increases the probability of getting infected. Besides, the risk of infection in people who ate cooked meat was 16 times lower than those who ate raw meat. The rest of the risk factors are listed in Table 1. As explored in our study, seropositivity was more prevalent in the center than in the north and south of West Azerbaijan province. Due to the use of a single locus (*GRA6*) for genotyping, there may be possibility of overlapping the sequence of the studied gene with other genotypes rather than type I. Therefore, we used *GRA6I* instead of type I in the text.

The results of the present study indicates that the seropositivity is significantly higher in pregnant women that ate undercooked meat and liver which can be explained by the interest of people in the area on eating undercooked barbecue and liver. Also, this area has a milder climate than the north and south of the aforementioned Province, which are mostly mountainous. There was no significant association between IgG and IgM seropositivity and place of residence, job, and education, which is similar to the findings reported by Hajsoleimani in Zanjan, the northwest of Iran [34].

Regarding the *T*. *gondii* isolated from abortion samples, they carried a shared sequence of *GRA6I*.

Among different markers, *GRA6*, a single-copy gene, was more polymorphic than others markers and could clearly differentiate into three different genotypes (I, II, and III) using a single PCR reaction, followed by single endonuclease (MseI) digestion [35]. PCR identifies an organism in many clinical specimens, including amniotic fluid, placenta, cerebrospinal fluid, and human blood. This technique provides rapid qualitative results with high sensitivity and specificity, especially when nested PCR is used [36, 37]. The identification of toxoplasmosis and genotyping *T*. *gondii* isolates is the key to the effective management of this infection in the rapid and accurate diagnosis of a disease [5, 16]. In Iran, the *T*. *gondii* types I, II, III were reported by Arefkhah et al. (2019) [26], Abdoli et al. (2017) [38], and Asgari et al. (2013) [39]. Type I had been recognized in a fatal CT case in Shiraz (south) [40] and also in ovine aborted fetuses in Qazvin (center) [41] and Khorasan Razavi (northeast) [35] Provinces of Iran. In the two latter studies, the same as our study, a history of consumption of undercooked meat was observed. In line with the present study, several investigations in Iran have reported the isolation of *T*. *gondii* DNA in the placenta of women with abortion. In one of these studies conducted in Shiraz, *T*. *gondii* was reported in 14.4% of paraffin-embedded blocks of aborted placenta, of which 54 (83.1%) cases had genotype II and 11 (16.9%) genotype III [39]. In another study in Ardabil, 16 (8%) patients had positive results in both ELISA and PCR on the fetuses [8]. In Iran, genotype III is the most prevalent type of *T*. *gondii* [42]; however, genotype II [14, 42] and in some studies genotype I have been reported in different hosts. In our study, *T*. *gondii GRA6I* was the most common among isolates [35, 43]. Type II genotype of *T*. *gondii* has been demonstrated to be responsible for most cases of CT in Europe and the United States [44]. In contrast to our finding, *Toxoplasma* type II is the most frequent in pregnant women with abortion in USA, France, Serbia, Romania, Argentina, and Portugal. Besides, type III, mix/recombinant, and atypical are the most prevalent types reported in France, Brazil, and Portugal. The type Africa I has also been reported in Turkey. *T*. *gondii* type I genotype, has been suggested to play a role in the development of CT in the USA, France, Tanzania, and Portugal [45]. In 2015 and 2017, two studies in Tehran could detect *T*. *gondii* type III among their samples using PCR. The former identified this type of *T*. *gondii* in 8 (3.8%) paraffin-embedded fetoplacental tissues and the latter in 9 (8.2%) aborted tissues [27, 38]. In the study of Eshratkhah Mohammadnejad et al. performed on pregnant women in Urmia, the molecular identification of *T*. *gondii* showed type I in isolates obtained from three newborns of IgM-positive mothers [27]. Two studies conducted in 2009 and 2012 by Tavassoli et al. in Urmia exhibited that *T*. *gondii* isolates from men and women were *T*. *gondii* type I genotype [46, 47]. Another study in Urmia reported toxoplasmosis in animals infected with *T*. *gondii* type I [25]. Therefore, in the studied area type I *T*. *gondii* is reported to be predominant, which is supported by the present study by detecting *GRA6I*-carrying strains in aborted fetuses in Urmia.

## 5. Conclusions

Taken together, in Iran, less attention has been paid to the destructive effects of toxoplasmosis in gynecological diseases. As more than 60% of the study population was negative for Toxoplasma’s specific antibody titers, this group is sensitive and at risk of infection. Based on the results of the present study, *T*. *gondii* is one of the causative agents of spontaneous abortion in West Azerbaijan, the northwest of Iran, and there is a link between toxoplasmosis and abortion. Moreover, *T*. *gondii GRA6I* are the major cause of *Toxoplasma*-related abortions in this province. Considering the data mentioned above, health specialists should pay much attention to toxoplasma. Screening *Toxoplasma* as a part of the TORCH test is suggested before and during pregnancy, which may save some unborn lives.

### 5.1 Limitations of study

The use of a single locus, the *GRA6* gene, was a major limitation in the present study, which may have overlapping sequences with different strains. Despite a 100% homology with Type I in GenBank, we did not indicate isolates as type I, instead we used strains carrying *GRA6I*.

## Supporting information

S1 Raw images Agarose gel electrophoresis of *T*. *gondii GRA6* gene amplification. (TIF)

S1 File Questionnaire used during sampling. (PDF)

S2 File Statistical analysis. (DOC)

S3 File Sequence alignment of GRA6I*(GRA6* allele of type I*)*. (PDF)
