# Supplementary figures and images for: Prevalence and risk factors of Toxoplasma gondii infection among women with miscarriage and their aborted fetuses in the northwest of Iran
Source: PLoS One. 2023 Oct 26;18(10):e0283493. doi: 10.1371/journal.pone.0283493 (PMC10602335; doi:10.1371/journal.pone.0283493)

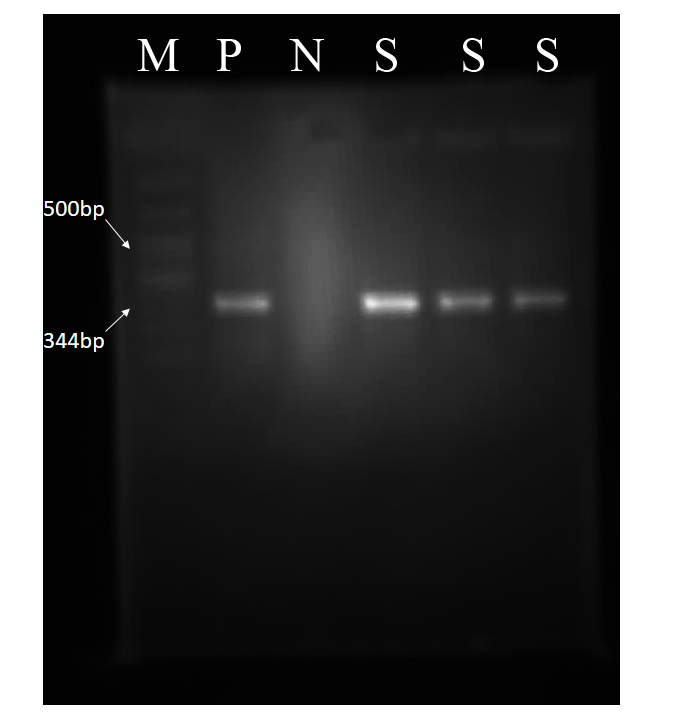

Supplement: S1 Raw images — (TIF) [file pone.0283493.s001.tif]
