# Supplementary material for: Prevalence and risk factors of Toxoplasma gondii infection among women with miscarriage and their aborted fetuses in the northwest of Iran
Source: PLoS One. 2023 Oct 26;18(10):e0283493. doi: 10.1371/journal.pone.0283493 (PMC10602335; doi:10.1371/journal.pone.0283493)
Supplement: S1 File — (PDF) [file pone.0283493.s002.pdf]

ID No. ___________

Prevalence and risk factors of *Toxoplasma gondii* infection among women with miscarriage and their aborted fetuses in the northwest of Iran

Main Researchers: Shiva Zeinali[1], Shahram Khademvatan[1]

Department of Medical Parasitology and Mycology& Cellular and Molecular Research Center, Cellular and Molecular Medicine Research Institute, Urmia University of Medical Sciences, Urmia, Iran

Please provide the necessary information or mark √ to applicable answers)
Name: ____
Address: ____

Age: _____ years
Civil Status: □ single □ married □ widowed

Highest Educational Attainment:
□ Elementary Level
□ High School level
□ Elementary Graduate
□ High School Graduate
□ College Level □ College Graduate
□ Others (please specify) ________________________

Job: ____

History of abortion □ No □ Yes
Present medical conditions: □ None □ Others (Please indicate)
Previous medical conditions/procedures: □ No □ yes

Do you eat raw foods (meat, Liver …)? □ No □ Yes (indicate where)
Do you drink un-boiled milk? □ No □ Yes
Washing of vegetable? □ No □ Water □ Detergent □ Salt
Soil contact : □ No □ Yes
Do you have constant contact with cats? □ No □ Yes (please describe frequency)
Do you own a cat? □ No □ Yes if yes □ Inside □Outside
____
