# Supplementary material for: Prevalence and risk factors of Toxoplasma gondii infection among women with miscarriage and their aborted fetuses in the northwest of Iran
Source: PLoS One. 2023 Oct 26;18(10):e0283493. doi: 10.1371/journal.pone.0283493 (PMC10602335; doi:10.1371/journal.pone.0283493)
Supplement: S2 File — (DOC) [file pone.0283493.s003.doc]

LOGISTIC REGRESSION VARIABLES toxoplasmaIgG
  /METHOD=BSTEP(LR) cat1 Soil2 Liver1 Vegetable2 age2 Job1 meat1 Home
  /CONTRAST (cat1)=Indicator
  /CONTRAST (Soil2)=Indicator
  /CONTRAST (meat1)=Indicator
  /CONTRAST (Liver1)=Indicator
  /CONTRAST (Vegetable2)=Indicator
  /CONTRAST (age2)=Indicator
  /CONTRAST (Job1)=Indicator
  /PRINT=CI(95)
  /CRITERIA=PIN(0.05) POUT(0.10) ITERATE(20) CUT(0.5).


Logistic Regression


Notes	
Output Created	24-SEP-2022 11:52:59	
Comments		
Input	Data	C:\Users\Rasool Jafari\OneDrive\stu thesis\Zeinali\SPSSTEZSHIVAZEINALI (asli)  14001207    14010209 (1)_11.sav	
	Active Dataset	DataSet1	
	Filter	<none>	
	Weight	<none>	
	Split File	<none>	
	N of Rows in Working Data File	215	
Missing Value Handling	Definition of Missing	User-defined missing values are treated as missing	
Syntax	LOGISTIC REGRESSION VARIABLES toxoplasmaIgG
  /METHOD=BSTEP(LR) cat1 Soil2 Liver1 Vegetable2 age2 Job1 meat1 Home
  /CONTRAST (cat1)=Indicator
  /CONTRAST (Soil2)=Indicator
  /CONTRAST (meat1)=Indicator
  /CONTRAST (Liver1)=Indicator
  /CONTRAST (Vegetable2)=Indicator
  /CONTRAST (age2)=Indicator
  /CONTRAST (Job1)=Indicator
  /PRINT=CI(95)
  /CRITERIA=PIN(0.05) POUT(0.10) ITERATE(20) CUT(0.5).	
Resources	Processor Time	00:00:00.06	
	Elapsed Time	00:00:00.19	


Case Processing Summary	
Unweighted Casesa	N	Percent	
Selected Cases	Included in Analysis	215	100.0	
	Missing Cases	0	.0	
	Total	215	100.0	
Unselected Cases	0	.0	
Total	215	100.0	

a. If weight is in effect, see classification table for the total number of cases.	


Dependent Variable Encoding	
Original Value	Internal Value	
neg	0	
pos	1	


Categorical Variables Codings	
	Frequency	Parameter coding	
		(1)	(2)	(3)	
age2	>20	26	1.000	.000	.000	
	21-30	113	.000	1.000	.000	
	31-40	75	.000	.000	1.000	
	<41	1	.000	.000	.000	
Vegetable2	Water	3	1.000	.000		
	Salt	76	.000	1.000		
	Detergent	136	.000	.000		
Job1	House	115	1.000	.000		
	Labor	41	.000	1.000		
	Staff	59	.000	.000		
Soil2	Lot
Intermediate	111	1.000	.000		
	Low	87	.000	1.000		
	no	17	.000	.000		
Liver1	Cooked	179	1.000			
	Juicy	36	.000			
meat1	Cooked	193	1.000			
	Juicy	22	.000			
cat1	Inside
Outside	116	1.000			
	no	99	.000			


Block 0: Beginning Block


Classification Tablea,b	
	Observed	Predicted	
		PosNeg	Percentage Correct	
		neg	pos		
Step 0	PosNeg	neg	145	0	100.0	
		pos	70	0	.0	
	Overall Percentage			67.4	

a. Constant is included in the model.	
b. The cut value is .500	


Variables in the Equation	
	B	S.E.	Wald	df	Sig.	Exp(B)	
Step 0	Constant	-.728	.146	25.037	1	.000	.483	


Variables not in the Equation	
	Score	df	Sig.	
Step 0	Variables	cat1(1)	46.020	1	.000	
		Soil2	40.829	2	.000	
		Soil2(1)	40.533	1	.000	
		Soil2(2)	26.393	1	.000	
		Liver1(1)	30.981	1	.000	
		Vegetable2	30.621	2	.000	
		Vegetable2(1)	1.612	1	.204	
		Vegetable2(2)	27.599	1	.000	
		age2	12.297	3	.006	
		age2(1)	11.313	1	.001	
		age2(2)	3.917	1	.048	
		age2(3)	.016	1	.898	
		Job1	1.685	2	.431	
		Job1(1)	1.680	1	.195	
		Job1(2)	.374	1	.541	
		meat1(1)	38.002	1	.000	
		Home	.405	1	.524	
	Overall Statistics	102.827	13	.000	


Block 1: Method = Backward Stepwise (Likelihood Ratio)


Omnibus Tests of Model Coefficients	
	Chi-square	df	Sig.	
Step 1	Step	125.707	13	.000	
	Block	125.707	13	.000	
	Model	125.707	13	.000	
Step 2a	Step	-.984	2	.611	
	Block	124.722	11	.000	
	Model	124.722	12	.000	
Step 3a	Step	-1.518	1	.218	
	Block	123.204	10	.000	
	Model	123.204	10	.000	

a. A negative Chi-squares value indicates that the Chi-squares value has decreased from the previous step.	


Model Summary	
Step	-2 Log likelihood	Cox & Snell R Square	Nagelkerke R Square	
1	145.625a	.443	.618	
2	146.610a	.440	.614	
3	148.128a	.436	.608	

a. Estimation terminated at iteration number 20 because maximum iterations has been reached. Final solution cannot be found.	


Classification Tablea	
	Observed	Predicted	
		PosNeg	Percentage Correct	
		neg	pos		
Step 1	PosNeg	neg	137	8	94.5	
		pos	15	55	78.6	
	Overall Percentage			89.3	
Step 2	PosNeg	neg	135	10	93.1	
		pos	14	56	80.0	
	Overall Percentage			88.8	
Step 3	PosNeg	neg	136	9	93.8	
		pos	18	52	74.3	
	Overall Percentage			87.4	

a. The cut value is .500	


Variables in the Equation	
	B	S.E.	Wald	df	Sig.	Exp(B)	95% C.I.for EXP(B)	
							Lower	Upper	
Step 1a	cat1(1)	1.956	.495	15.633	1	.000	7.070	2.681	18.640	
	Soil2			12.052	2	.002				
	Soil2(1)	2.841	1.417	4.019	1	.045	17.140	1.066	275.702	
	Soil2(2)	1.392	1.448	.925	1	.336	4.025	.236	68.764	
	Liver1(1)	-1.563	.619	6.384	1	.012	.209	.062	.704	
	Vegetable2			9.292	2	.010				
	Vegetable2(1)	2.403	2.534	.900	1	.343	11.057	.077	1585.701	
	Vegetable2(2)	1.292	.435	8.820	1	.003	3.638	1.551	8.533	
	age2			7.159	3	.067				
	age2(1)	16.657	40193.460	.000	1	1.000	17135471.979	.000	.	
	age2(2)	15.053	40193.460	.000	1	1.000	3448038.902	.000	.	
	age2(3)	15.720	40193.460	.000	1	1.000	6717223.567	.000	.	
	Job1			.975	2	.614				
	Job1(1)	-.526	.574	.839	1	.360	.591	.192	1.821	
	Job1(2)	-.206	.702	.086	1	.769	.814	.206	3.220	
	meat1(1)	-2.741	.911	9.054	1	.003	.064	.011	.385	
	Home	.380	.512	.552	1	.458	1.462	.536	3.987	
	Constant	-16.752	40193.460	.000	1	1.000	.000			
Step 2a	cat1(1)	1.958	.492	15.846	1	.000	7.086	2.702	18.584	
	Soil2			12.438	2	.002				
	Soil2(1)	2.975	1.462	4.139	1	.042	19.593	1.115	344.267	
	Soil2(2)	1.534	1.492	1.057	1	.304	4.635	.249	86.338	
	Liver1(1)	-1.506	.605	6.193	1	.013	.222	.068	.726	
	Vegetable2			9.372	2	.009				
	Vegetable2(1)	2.404	2.416	.990	1	.320	11.066	.097	1259.334	
	Vegetable2(2)	1.285	.432	8.854	1	.003	3.614	1.551	8.425	
	age2			7.073	3	.070				
	age2(1)	16.579	40193.950	.000	1	1.000	15861141.508	.000	.	
	age2(2)	15.033	40193.950	.000	1	1.000	3379920.067	.000	.	
	age2(3)	15.757	40193.950	.000	1	1.000	6966390.064	.000	.	
	meat1(1)	-2.821	.914	9.521	1	.002	.060	.010	.357	
	Home	.548	.449	1.494	1	.222	1.730	.718	4.168	
	Constant	-17.424	40193.950	.000	1	1.000	.000			
Step 3a	cat1(1)	2.033	.488	17.377	1	.000	7.634	2.936	19.851	
	Soil2			11.583	2	.003				
	Soil2(1)	2.929	1.421	4.245	1	.039	18.703	1.153	303.269	
	Soil2(2)	1.624	1.451	1.253	1	.263	5.073	.295	87.098	
	Liver1(1)	-1.481	.593	6.228	1	.013	.227	.071	.728	
	Vegetable2			8.972	2	.011				
	Vegetable2(1)	2.114	2.346	.812	1	.368	8.280	.083	821.700	
	Vegetable2(2)	1.248	.427	8.552	1	.003	3.482	1.509	8.034	
	age2			7.736	3	.052				
	age2(1)	16.921	40192.393	.000	1	1.000	22319040.171	.000	.	
	age2(2)	15.291	40192.393	.000	1	1.000	4372717.195	.000	.	
	age2(3)	15.943	40192.393	.000	1	1.000	8389914.930	.000	.	
	meat1(1)	-2.894	.919	9.921	1	.002	.055	.009	.335	
	Constant	-16.828	40192.393	.000	1	1.000	.000			

a. Variable(s) entered on step 1: cat1, Soil2, Liver1, Vegetable2, age2, Job1, meat1, Home.	


Model if Term Removed	
Variable	Model Log Likelihood	Change in -2 Log Likelihood	df	Sig. of the Change	
Step 1	cat1	-82.086	18.547	1	.000	
	Soil2	-79.709	13.792	2	.001	
	Liver1	-76.208	6.791	1	.009	
	Vegetable2	-77.637	9.648	2	.008	
	age2	-76.585	7.545	3	.056	
	Job1	-73.305	.984	2	.611	
	meat1	-78.974	12.323	1	.000	
	Home	-73.090	.554	1	.457	
Step 2	cat1	-82.690	18.771	1	.000	
	Soil2	-80.561	14.512	2	.001	
	Liver1	-76.563	6.517	1	.011	
	Vegetable2	-78.195	9.780	2	.008	
	age2	-77.017	7.424	3	.060	
	meat1	-79.824	13.039	1	.000	
	Home	-74.064	1.518	1	.218	
Step 3	cat1	-84.560	20.992	1	.000	
	Soil2	-80.754	13.379	2	.001	
	Liver1	-77.335	6.542	1	.011	
	Vegetable2	-78.706	9.285	2	.010	
	age2	-78.110	8.093	3	.044	
	meat1	-81.043	13.959	1	.000	


Variables not in the Equation	
	Score	df	Sig.	
Step 2a	Variables	Job1	.982	2	.612	
		Job1(1)	.897	1	.343	
		Job1(2)	.139	1	.709	
	Overall Statistics	.982	2	.612	
Step 3b	Variables	Job1	1.956	2	.376	
		Job1(1)	1.476	1	.224	
		Job1(2)	.011	1	.917	
		Home	1.509	1	.219	
	Overall Statistics	2.502	3	.475	

a. Variable(s) removed on step 2: Job1.	
b. Variable(s) removed on step 3: Home.	
