# Supplementary material for: Prevalence and risk factors of Toxoplasma gondii infection among women with miscarriage and their aborted fetuses in the northwest of Iran
Source: PLoS One. 2023 Oct 26;18(10):e0283493. doi: 10.1371/journal.pone.0283493 (PMC10602335; doi:10.1371/journal.pone.0283493)
Supplement: S3 File — (PDF) [file pone.0283493.s004.pdf]

Sequences alignment of Isolates and references

>Isolate 194

CGTAGCGTGCTTGTTGGCGACTACCTTTTTTTCTTGGGAGTGTCGGCGAAATGGCACACGGTGGCATCCATCTGAG
GCAGAAGCGTAACTTCTGTCCTGTAACTGTCTCCACAGTTGCTGTGGTCTTTGTAGTCTTCATGGGTGTACTCGTCA
ATTCGTTGGGTGGAGTCGCTGTCGCAGCAGACAGCGGTGGTGTTAAGCAGACCCCTTCGGAAACCGGTTCGAGCG
GTGGACAGCAAGAAGCAGTGGGGACCACTGAAGACTATGTCAACTCTTCGGCGAA

>Isolate 195

CGTAGCGTGCTTGTTGGCGACTACCTTTTTTTCTTGGGAGTGTCGGCGAAATGGCACACGGTGGCATCCATCTGAG
GCAGAAGCGTAACTTCTGTCCTGTAACTGTCTCCACAGTTGCTGTGGTCTTTGTAGTCTTCATGGGTGTACTCGTCA
ATTCGTTGGGTGGAGTCGCTGTCGCAGCAGACAGCGGTGGTGTTAAGCAGACCCCTTCGGAAACCGGTTCGAGCG
GTGGACAGCAAGAAGCAGTGGGGACCACTGAAGACTATGTCAACTCTTCGGCGAA

>Isolate 72

CGTAGCGTGCTTGTTGGCGACTACCTTTTTTTCTTGGGAGTGTCGGCGAAATGGCACACGGTGGCATCCATCTGAG
GCAGAAGCGTAACTTCTGTCCTGTAACTGTCTCCACAGTTGCTGTGGTCTTTGTAGTCTTCATGGGTGTACTCGTCA
ATTCGTTGGGTGGAGTCGCTGTCGCAGCAGACAGCGGTGGTGTTAAGCAGACCCCTTCGGAAACCGGTTCGAGCG
GTGGACAGCAAGAAGCAGTGGGGACCACTGAAGACTATGTCAACTCTTCGGCGAA

>Type I

CGTAGCGTGCTTGTTGGCGACTACCTTTTTTTCTTGGGAGTGTCGGCGAAATGGCACACGGTGGCATCCATCTGAG
GCAGAAGCGTAACTTCTGTCCTGTAACTGTCTCCACAGTTGCTGTGGTCTTTGTAGTCTTCATGGGTGTACTCGTCA
ATTCGTTGGGTGGAGTCGCTGTCGCAGCAGACAGCGGTGGTGTTAAGCAGACCCCTTCGGAAACCGGTTCGAGCG
GTGGACAGCAAGAAGCAGTGGGGACCACTGAAGACTATGTCAACTCTTCGGCGAA

>Type II

CGTAGCGTGCTTGTTGGCGACTACCTTTTTTTCTTGGGAGTGTCGGCGAAATGGCACACGGTGGCATCTATCTGAG
GCAGAAGCGTAACTTCTGTCCTTTAACTGTCTCCACAGTTGCTGTGGTCTTTGTAGTCTTCATGGGTGTACTCGTCA
ATTCGTTGGGTGGAGTCGCTGTCGCAGCAGACAGCGGTGGTGTTAGGCAGACCCCTTCGGAAACCGGTTCGAGC
GGTGGACAGCAAGAAGCAGTGGGGACCACTGAAGACTATGTCAACTCTTCGGCGAA

>Type III

CGTAGCGTGCTTGTTGGCGACTACCTTTTTTTCTTGGGAGTGTCGGCGAAATGGCACACGGTGGCATCCATCTGAG
GCAGAAGCGTAACTTCTGTCCTTTAACTGTCTCCACAGTTGCTGTGGTCTTTGTAGTTTTCATGGGTGTACTCGTCA
ATTCGTTGGGTGGAGTCGCTGTCGCAGCAGACAGCGATGGTGTTAAGCAGACCCCTTCGGAAACCGGTTCGAGCG
GTGGACAGCAAGAAGCAGTGGGGACCACTGAAGACTATGTCAACTCTTCGGCGAA
